# In Vitro Anticancer Activity of Novel Ciprofloxacin Mannich Base in Lung Adenocarcinoma and High-Grade Serous Ovarian Cancer Cell Lines via Attenuating MAPK Signaling Pathway

**DOI:** 10.3390/molecules28031137

**Published:** 2023-01-23

**Authors:** Michael A. Fawzy, Rania H. Abu-baih, Gamal El-Din A. Abuo-Rahma, Islam M. Abdel-Rahman, Azza A. K. El-Sheikh, Maiiada H. Nazmy

**Affiliations:** 1 Department of Biochemistry, Faculty of Pharmacy, Minia University, Minia 61519, Egypt; 2 Department of Medicinal Chemistry, Faculty of Pharmacy, Minia University, Minia 61519, Egypt; 3 Department of Pharmaceutical Chemistry, Faculty of Pharmacy, Deraya University, New-Minia, Minia 61511, Egypt, Egypt; 4 Basic Health Sciences Department, College of Medicine, Princess Nourah bint Abdulrahman University, P.O. Box 84428, Riyadh 11671, Saudi Arabia

**Keywords:** ciprofloxacin, drug repositioning, cancer, apoptosis, cell cycle, MAPK pathway, P53/Bax/Bcl2 pathway

## Abstract

Novel drugs are desperately needed in order to combat a significant challenge due to chemo-therapeutic resistance and bad prognosis. This research aimed to assess the anticancer activity of a newly synthesized ciprofloxacin Mannich base (CMB) on ovarian cancer (OVCAR-3) and lung cancer (A-549) cell lines and to investigate probable involved molecular mechanisms. The cytotoxic and pro-apoptotic impact of CMB on both cell lines was investigated using MTT assay, Annexin V assay, and cell cycle analysis, as well as caspase-3 activation. Western blotting was carried out to evaluate downstream targets of the MAPK pathway, while qRT PCR was used to evaluate the gene expression pattern of the p53/Bax/Bcl2 pathway. CMB treatment showed significantly reduced cell proliferation in both OVCAR-3 and A-549 cells with half maximum inhibitory concentration (IC_50_) = 11.60 and 16.22 µg/mL, respectively. CMB also induced apoptosis, S phase cell cycle arrest, and up-regulated expression of p53, p21, and Bax while down-regulated Bcl2 expression. CMB also halted cell proliferation by deactivating the MAPK pathway. In conclusion, CMB may be regarded as a potential antiproliferative agent for lung and ovarian cancers due to anti-proliferative and pro-apoptotic actions via inhibition of the MAPK pathway and p53/Bax/Bcl2.

## 1. Introduction

During decades, carcinogenesis has evolved into a multi-step complex, stochastic, and highly coordinated process [1]. Regarding the American Cancer Society, around 8.2 million deaths and roughly 14.1 million new cases of cancer have been documented [2]. Lung carcinoma is the most prevalent and invasive malignant tumor accounting for nearly one million deaths worldwide [3]. According to statistics, non-small-cell lung cancer (NSCLC) affects eighty-five percent of lung cancer patients overall and accounts for 80 percent of lung cancer mortality [4]. Smoking, occupational toxins, and carcinogens are key factors attributing to NSCLC development and progression [5,6]. Ovarian cancer represents the worst gynecological malignancy. Epithelial ovarian cancers (EOC) are a diverse set of disorders that, based on histology and molecular genetics, may be categorized into five major types: endometrioid tumors, mucinous tumors, clear cell tumors, low-grade serous tumors, and High-grade serous tumors [7]. High-grade serous ovarian cancer (HGSOC) represents the foremost common and lethal type of EOC due to late diagnosis [8]. HGSCs are among the most genetically modified malignancies, with rare mutations and substantial somatic copy number changes [9,10].

Multiple pathways have been reported to be implicated in regulating lung and ovarian cancer proliferation. Proliferation, migration, invasion, and chemotherapy resistance are fundamentally regulated by mitogen-activated protein kinase (MAPK) pathways [11,12]. To transduce extracellular signals into the nucleus, three different MAPK pathways have been elucidated. Extracellular signal-regulated kinase (ERK), c-Jun NH2-terminal kinase (JNK), and p38 are examples of these pathways. G-protein-coupled receptors and receptor tyrosine kinase, for example, are cell surface receptors that transmit signals that activate the MAPK/ERK pathway. Improper pathway regulation results in aberrant cellular activity, which promote carcinogenesis by increasing proliferation, cell growth, survival, and de-differentiation [13]. Following MAPK/ERK activation, the upstream RAS is activated, which in turn draws RAFs to the cell membrane, where they phosphorylate MEK1/2 and activate ERK1/2 [14]. Phosphorylated ERK1/2 induce RSK phosphorylation, then both phosphorylated ERK1/2 and RSK are transported to the nucleus, where they promote phosphorylation of numerous transcription factors, including c-Jun, c-Myc, Elk-1, c-Fos, and activating transcription factor 2 (ATF2), subsequently promoting cell progression [15].

The JNK pathway is another crucial MAPK pathway that is concerned with the regulation of inflammatory responses, apoptosis, cellular growth, and cancer development [16]. JNK activity is dependent on upstream MKK4 and MKK7, which are triggered as a result of stress signals and inflammatory cytokines [17]. JNKs phosphorylate and induce activation of a variety of non-nuclear and nuclear proteins, such as c-Myc, Elk1, transcription factor activator protein-1, NFAT, A,TF-2, and P53 [18]. JNK’s downstream targets include c-Jun, which is phosphorylated by JNK and then translocated to the nucleus. Furthermore, c-Jun is well known for regulating pro-apoptotic or anti-apoptotic gene expression, including B-cell lymphoma-2 (Bcl-2) and Bcl-2 Associated X-Protein (BAX) [19].

Growing evidence shows that ERK/MAPK and JNK/MAPK signaling dysregulation causes numerous alterations in gene expressions involved in cell cycle, migration, differentiation, and cancer stem cell; all of these are important for NSCLC and HGSOC pathogenesis and treatment resistance [20,21,22,23,24,25,26].

Apoptosis is one of the main anticancer therapy mechanisms [27]. Extrinsic and intrinsic mitochondrial deaths are the two most common types of apoptosis. Many reports have demonstrated that the latter is the most prevalent one and has a vital role in apoptosis precision [28]. Cell cycle, DNA repair, aging, and apoptosis are all regulated by transcription factor p53 [29]. P53 is phosphorylated and acetylated by posttranslational modification in response to various stressors such as DNA damage, then translocated to the nucleus to trans-activate multiple genes, which control DNA repair, apoptosis, and cell cycle arrest. Additionally, TP53 mutations resulted in the acquisition of extra oncogenic capabilities such as survival and growth, besides the loss of cancer suppressive functions [30]. The most predominant target genes of p53 are Bax and p21 [31]. P21 blocks the activity of cyclin-dependent kinase, which impairs cell cycle evolution leading to DNA repair and cell cycle arrest. P53 promotes apoptosis through the intrinsic pathway by controlling Bax and Bcl2 genes’ transcription [32]. The Bcl2 family antiapoptotic members control apoptosis by acting as anti-pore mediators in intrinsic apoptosis [33]. Meanwhile, the pro-apoptotic Bax assists in the creation of holes in mitochondrial membranes and determines whether or not apoptosis will occur [34].

Due to the high costs and increased failure rates of developing novel anticancer medicines for clinical therapy, repositioning existing pharmaceuticals and reevaluating them for new biological activity is the most convenient option since it involves less time and money. Ciprofloxacin (CIP) is a second-generation fluoroquinolone with significant anti-bacterial activity, several therapeutic applications, and minimal side effects [35]. Despite having an antibacterial effect, CIP is also noted for its cytotoxic activity, which is mediated via inducing DNA damage and cell cycle arrest leading to apoptosis. Moreover, CIP has been shown to display antitumor and pro-apoptotic effects against many cancer cell lines [36]. For all these reasons, efforts are now made to design new CIP derivatives with enhanced cytotoxicity. One of the most recent rationales designed CIP derivative is ciprofloxacin Mannich base (CMB) with enhanced cytotoxicity against various cancer cell lines. A new C7-piperazinyl ciprofloxacin ortho-phenolic Mannich base of ciprofloxacin X was synthesized and identified using spectroscopic and elemental analysis with the goal of improving their physicochemical qualities, which may have an effect on their anti-cancer activity. The naphthol Mannich bases X showed exceptional cytotoxic activity toward 60 tested cancer cells [37].

The current study sought to elucidate a putative molecular mechanism behind the antitumor activity of CMB in OVCAR-3 and A-549 cell lines to offer theoretical and experimental basis for clinical applications.

## 2. Results

### 2.1. Cell Viability Assay

**Figure 1:**
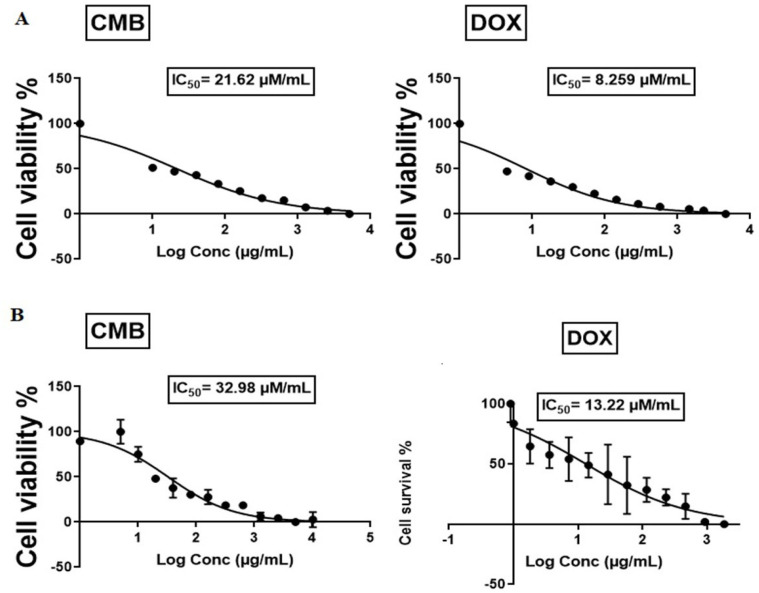
Cytotoxic effects of CMB and doxorubicin treatment in OVCAR−3 and A−549 cell lines. (**A**) OVCAR-3 cell lines. (**B**) A−549 cell lines. Results from MTT test obtained after a 48−h incubation period with both treatments. Means and SE were calculated from three independent experiments.

The cytotoxic activity of CMB on human OVCAR-3 and A-549 cells was assessed using an MTT assay, with doxorubicin serving as a reference drug. CMB, as demonstrated in Figure 1, attenuated cell proliferation in a concentration-dependent approach after 48 h. The cells’ survival was denoted as a percent relative to control cells. The results elucidated that OVCAR-3 cells were more sensitive to CMB, with a lower half maximum inhibitory concentration (IC_50_) value (21.62 µM/mL) than A-549 cells (32.98 µM/mL) (Figure 1).

### 2.2. CMB Induced Apoptosis in OVCAR-3 and A-549 Cell Lines

**Figure 2:**
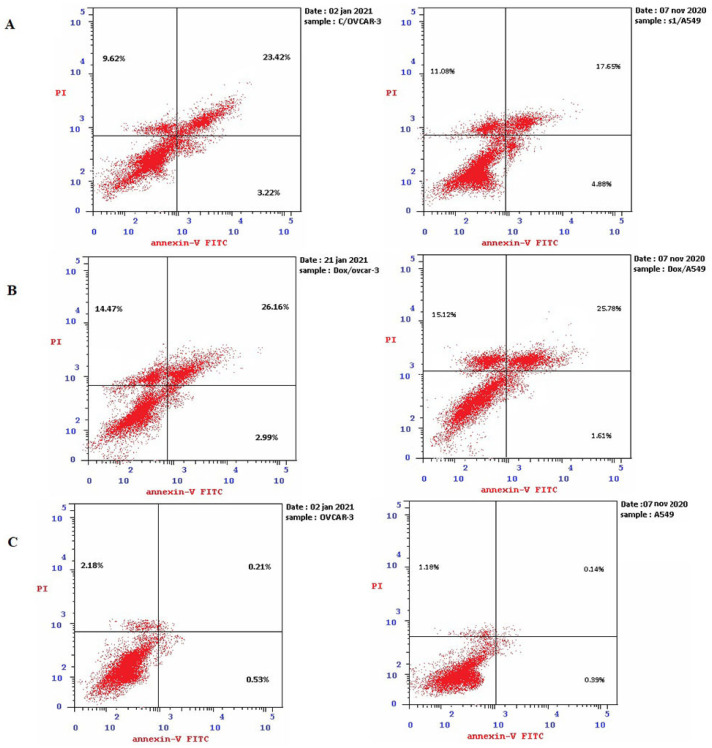
Cell apoptosis analysis of CMB and doxorubicin treatment on OVCAR−3 and A−549 cells after incubation for 48 h. The PI staining intensity is shown on the y-axis in log units, and the FITC-Annexin V staining intensity is shown on the x-axis. Lower left (LL) quadrant of a dot or density diagram represents normal viable cells. Upper right (UR) quadrant of a dot or density map represents late apoptotic cells. Lower right (LR) quadrant of a dot or density map contains early apoptotic cells. Necrotic cells are found in the dot’s upper left (UL) quadrant. (**A**): OVCAR−3 and A−549 cells that have received CMB treatment (IC_50_). (**B**): OVCAR−3 and A−549 cells treated with doxorubicin (IC_50_). (**C**): OVCAR−3 and A−549 control cells with no treatment.

OVCAR-3 and A-549 cells were subjected to an annexin V-FITC/PI dual staining test. It has been revealed that, following 48 h of CMB treatment (IC_50_), the percentage of total apoptotic cells was raised from 2.92% (control cells) to 36.26% in OVCAR-3 cells and from 1.71% (control cells) to 33.61% in A-549 cells. The percent of early apoptotic cells raised from 0.53% (control) to 3.22% (CMB treated cells) in OVCAR-3 cells and from 0.39% (con-trol) to 4.88% (CMB treated cells) in A-549 cells. In addition, the percent of the late apoptotic population raised from 0.21% (control) to 23.4% (CMB treated cells) in OVCAR-3 cells and from 0.14% (control) to 17.65% (CMB treated cells) in A-549 cells (Figure 2 and Table 1).

### 2.3. CMB Induced S Phase Cell Cycle Arrest in OVCAR-3 and A-549 Cells

**Figure 3:**
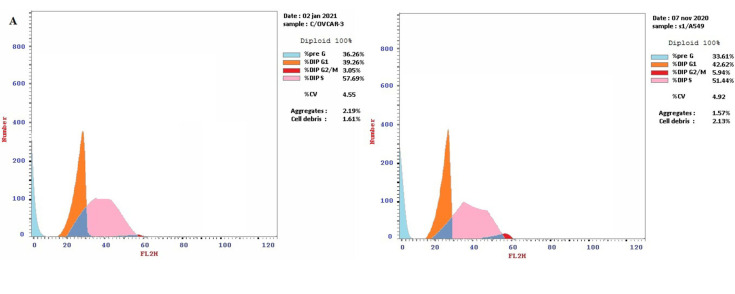

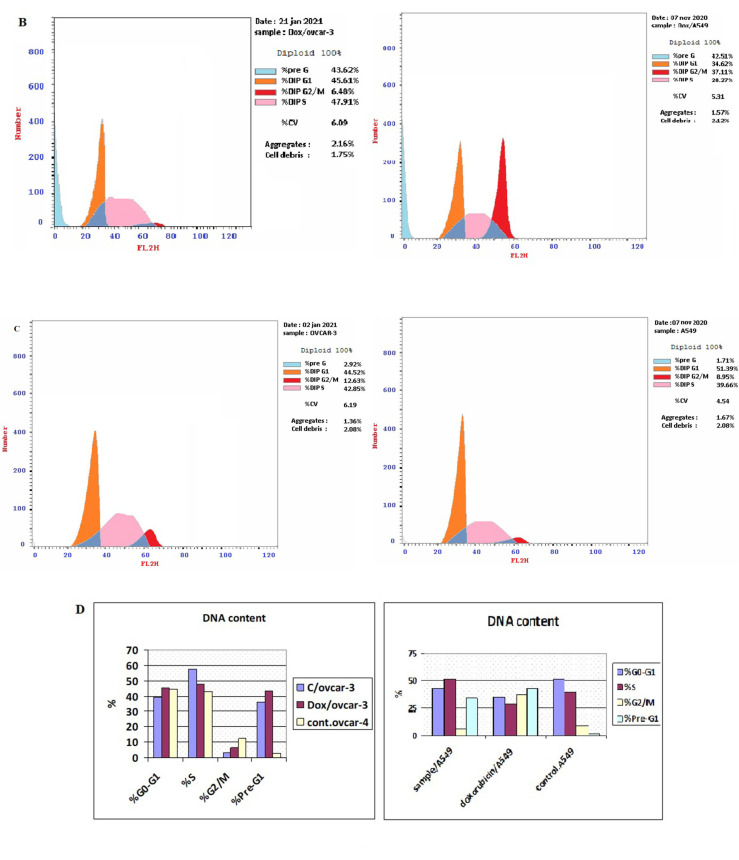
Cell cycle analysis of CMB and Doxorubicin treated OVCAR−3 and A−549 cells: (**A**): Treatment of OVCAR−3 and A−549 cells with CMB (IC_50_) for 48 h. (**B**): Treatment of OVCAR−3 and A−549 cells with doxorubicin (IC_50_) for 48 h. (**C**): OVCAR−3 and A−549 untreated control cells. (**D**): DNA content in each stage of cell cycle following treatment with CMB and doxorubicin (IC_50_) for 48 h.

The cell cycle pattern altered dramatically in all treated cells when compared with the untreated control (Figure 3). Following treatment with CMB, the cell proportion at the G2/M phase decreased to 3.05% and 5.94% in OVCAR-3 and A-549 cells, respectively. In addition, the cell proportion at the S phase increased to 57.69% and 51.44% in OVCAR-3 and A-549 cells, respectively, as compared to control cells. The altered cell cycle pattern strongly implied that CMB induced S phase cell cycle arrest. In addition, the pre-G1 population was raised to 36.26% and 33.61% in OVCAR-3 and A-549 cells, respectively, following treatment with CMB (2.92% and 1.71% in the control cells). The presence of a pre-G1 peak (apoptotic cells) observed in OVCAR-3, and A-549 cells treated with CMB reconfirmed the apoptosis assay data above.

### 2.4. Gene Expression Analysis of p53/Bax/Bcl2 and p21 Signaling Pathway in OVCAR-3 and A-549 Cell Lines

**Figure 4:**
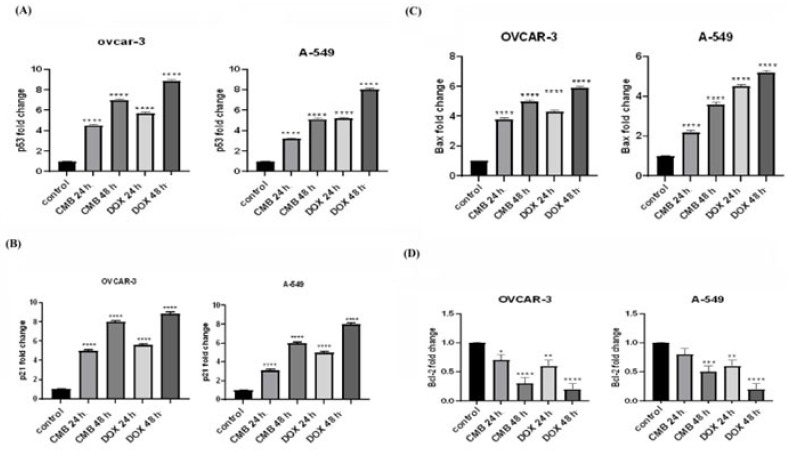
Molecular evaluation of gene expression in OVCAR-3 and A-549 cells after incubation with CMB or doxorubicin IC_50_ values for 24 h or 48 h. Relative gene expression is represented as mean ± SEM. (**A**) P53 gene expression in OVCAR-3 and A-549 cells. (**B**) P21 gene expression in OVCAR-3 and A-549 cells. (**C**) Bax mRNA level in OVCAR-3 and A-549 cells. (**D**) Bcl2 mRNA level in OVCAR-3 and A-549 cells. Means and SE were calculated from three independent experiments. Statistically significant results compared to untreated control were displayed as: * *p* < 0.05; ** *p* < 0.01; *** *p* < 0.001; **** *p* < 0.0001.

The OVCAR-3 and A-549 cell lines were treated for 24 h or 48 h with the IC_50_ dose of CMB, with doxorubicin serving as a reference (Figure 4). Treatment of OVCAR-3 cells with CMB for 24 h or 48 h significantly upregulated the gene expression of p53 in a time- dependent way and led to a 4.5- and 7.01-fold increase, respectively, in comparison with untreated control cells (*p* < 0.001). In addition, treating A-549 cells with CMB for 24 h or 48 h significantly upregulated the gene transcription of p53 in a time dependent fashion and led to a 3.2- and 5.1-fold increase, respectively, relative to untreated control cells and after normalization to GAPDH (Figure 4A).

Subsequently, we analyzed the gene expression of p21. Our results revealed a significant time dependent upregulation in p21 gene expression in OVCAR-3 cells in response to CMB treatment for 24 h or 48 h with a 5- and 8-fold increase, respectively, when com-pared to the relative expression of the p21 gene in untreated control cells (*p* < 0.001). While in A-549 cells, gene expression of p21 was significantly upregulated in a time-dependent manner with to 3- and 6-fold increase, respectively, when compared to the relative gene expression of p21 in untreated control cells and after normalization to GAPDH (Figure 4B).

CMB treatment for 24 h or 48 h significantly upregulated Bax mRNA levels in OVCAR-3 cells (3.8- and 5.06-fold increase) relative to untreated control cells (*p* < 0.05). Furthermore, CMB treatment for 24 h or 48 h caused a substantial upregulation of Bax gene expression in A-549 cells (2.25- and 3.63-fold increase) in comparison with untreated control cells and after normalization to GAPDH (*p* < 0.05) (Figure 4C).

Furthermore, the relative gene expression of Bcl-2 was significantly downregulated in OVCAR-3 cancer cells treated with CMB for 24 h or 48 h to 0.70 and 0.33, respectively, compared to untreated control cells. Regarding A-549 cells, it was observed that Bcl2 gene expression was significantly downregulated after treatment with CMB for 24 h or 48 h to 0.81 and 0.51, respectively, compared to untreated control cells and after normalization to GAPDH (Figure 4D).

Interestingly, CMB induced a time-dependent rise in the Bax/Bcl-2 ratio in both OVCAR-3 and A-549 cells. Therefore, our data revealed that CMB promoted cell apoptosis through the intrinsic pathway (Figure 4E).

### 2.5. CMB Attenuates Tumorigenesis via Modulating the Ras/Raf/Mek/Erk Signaling Pathway

**Figure 5:**
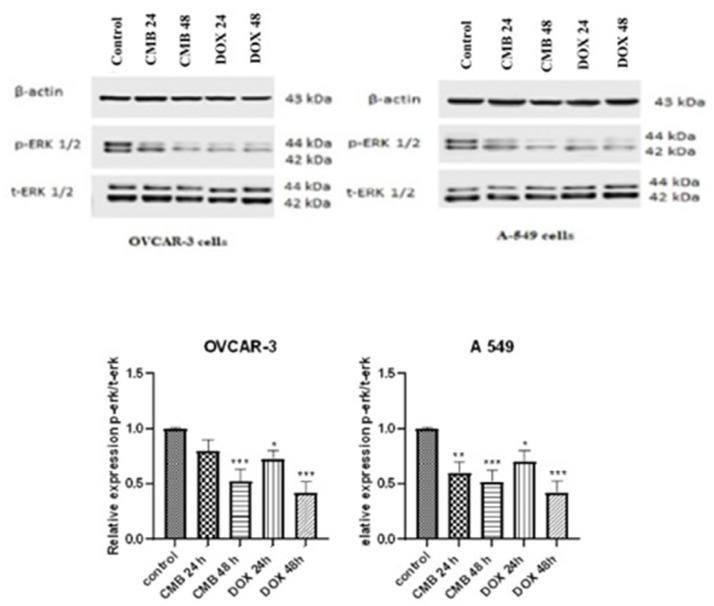
Western blots of ERK1/2 protein expression in OVCAR-3 and A-549 cells incubated with CMB and doxorubicin at IC_50_ values for 24 h or 48 h. Means and SE were calculated from three independent experiments. Statistically significant results relative to untreated control were presented as: * *p* < 0.05; ** *p* < 0.01; *** *p* < 0.001.

Western blot results indicated high levels of p-ERK1/2 in OVCAR-3 and A-549 cells. However, treatment with CMB for 24 h or 48 h suppressed the expression levels of p-ERK/t-ERK in OVCAR-3 cells to 0.8 and 0.5, respectively, in a time-dependent fashion relative to untreated control cells. Regarding A-549 cells, the protein level of p-ERK1/2 was significantly elevated. More interestingly, CMB treatment for 24 h or 48 h markedly suppressed the p-ERK1/2/t-ERK1/2 to 0.6 and 0.5, respectively, in a time-dependent way compared to untreated control cells (Figure 5).

### 2.6. CMB Attenuates Tumorigenesis via Modulating the Mkk4/7/Jnk Pathway

**Figure 6:**
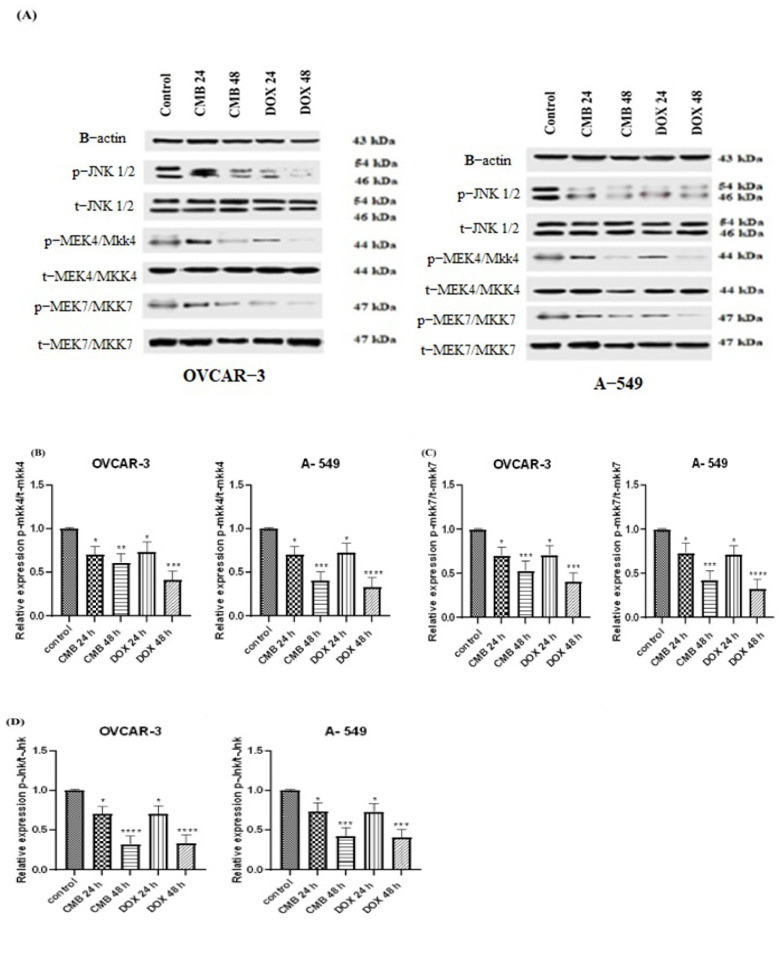
CMB suppresses the protein expression levels of p−mkk4, p−mkk7, and p−JKN1/2. (**A**–**D**) Western blot analysis of the protein levels of p−mkk4, p−mkk7, and p−JKN1/2 in OVCAR-3 and A-549 cells, respectively, incubated with CMB and doxorubicin at their IC_50_ values for 24 h or 48 h. Means and SE were calculated from three independent experiments. Statistically significant results relative to untreated control were displayed as: * *p* < 0.05; ** *p* < 0.01; *** *p* < 0.001; **** *p* < 0.0001.

As illustrated in Figure 6, a notable time-dependent suppression of the protein levels of p-mkk4/t-mkk4 (0.7–0.6) and p-mkk7/t-mkk7 (0.7–0.5) was seen after treating OVCAR-3 cells for 24 h or 48 h with the IC_50_ dose of CMB when compared to untreated control cells. Moreover, our data revealed a significant time-dependent suppression in the protein level of p-Jnk1/2/t–Jnk1/2 (0.6–0.3) after CMB treatment for 24 h or 48 h, respectively, when relative to untreated control cells.

Concerning A-549 cells, CMB treatment for 24 h or 48 h was capable of robustly inhibiting the protein level of p-mkk4/t–mkk4 (0.7–0.4) and p-mkk7/t–mkk7 (0.7–0.3), respectively, in a time-dependent fashion in comparison with untreated control cells. More interestingly, our data revealed a robust time dependent suppression in the protein level of p-Jnk1/2/t–Jnk1/2 (0.7–0.4) after CMB treatment for 24 h or 48 h, respectively, relative to untreated control cells.

### 2.7. Assessment of Caspase-3 Activation in OVCAR-3 and A-549 Cancer Cells

**Figure 7:**
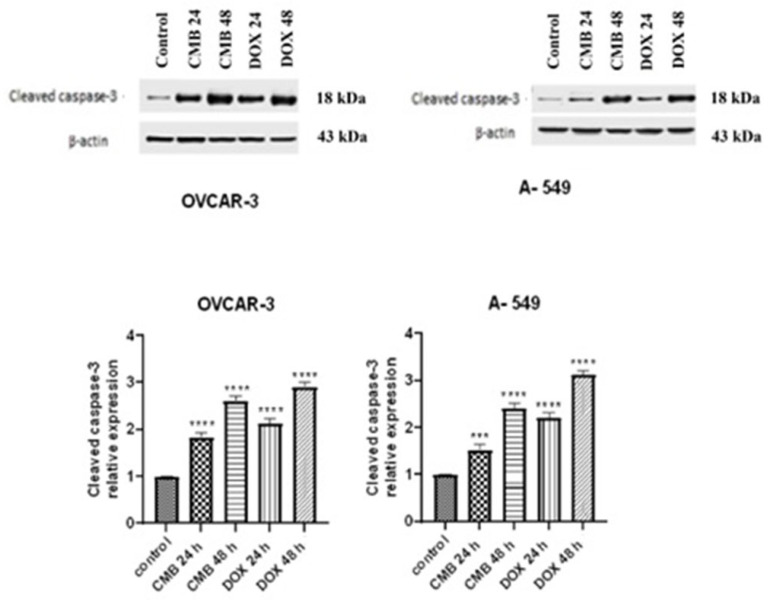
Western blots of active caspase-3 levels in OVCAR-3 and A-549 cells, respectively, incubated with CMB and doxorubicin at IC_50_ values for 24 h or 48 h. Means and SE were calculated from three independent experiments. Statistically significant results compared to untreated control were presented as: *** *p* < 0.001; **** *p* < 0.0001.

CMB treatment for 24 h or 48 h caused robust activation of caspase-3 in OVCAR-3 cells, reflecting a rise in active caspase-3 expression by 1.8 and 2.6 folds, respectively, in a time-dependent manner in comparison with untreated control cells (Figure 7). Similar observations were noticed in A-549 cells. Here, significant time-dependent elevation was observed in active caspase-3 level in A-549 as evidenced by 1.5 and 2.4 folds, in comparison with untreated control cells, which ascertained that CMB triggered apoptosis in OVCAR-3 and A-549 cancer cells via caspase-3 activation.

## 3. Discussion

Despite numerous therapeutic possibilities, such as surgical procedures combined with chemotherapy, immunotherapy, and radiotherapy, several deaths linked to lung and ovarian cancers are nevertheless noticeable. The announced mortalities induced by these malignancies represent 25% of recently diagnosed lung and 5% of ovarian cases [38]. This fact might be due to the ineffectiveness of therapies utilized supplemental to the surgical techniques. Consequently, developing new supportive therapies at the earliest stage of the disease is critical to reducing the frequency of relapses and enhancing therapeutic outcomes. Numerous reports have demonstrated the antiproliferative and anticancer potential of several fluoroquinolones against different cancer cell lines [37]. Fluoroquinolones are synthetic antibiotics with a broad-spectrum activity that are frequently used against many diseases. In several cancer cell lines, certain members show anticancer activity, making them exceptional among other antibiotics classes [39,40]. This anticancer effect was attributed to its ability to suppress the eukaryotic topoisomerase II enzyme resulting in mitochondrial DNA synthesis inhibition, mitochondrial damage, respiratory chain inhibition, and ATP deficiency. Energy deficiency promotes apoptosis by causing G2/M and/or S phase cell cycle arrest [41].

Our current research investigated the effect of a novel CMB on the viability of OVCAR-3 and A-549 cells. Several investigations have shown that the parent medication CIP has a cytotoxic impact on certain cancer cells. However, the observed cytotoxic effect in different types of cancers was minimal. Therefore, the CIP structure was altered and modified in order to boost its cytotoxicity and anti-proliferative activity. This is consistent with earlier findings that were reported by Eslam et al., who confirmed that substituting the CIP core at N-4 of piperazine with a structural motif containing a fused benzene ring with an o-hydroxyl substitution on the phenyl ring led to potential antiproliferative activities [37].

Our data showed that CMB suppressed OVCAR-3 and A-549 viability in a concentration-dependent manner with preferential cytotoxicity toward ovarian cancer cells over lung cancer cells. This enhanced cytotoxicity could be attributed to the hypothesis that the inclusion of the N-4-piperazinyl Mannich base naphthyl group lowered the water solubility of CIP by altering the zwitter ionic nature of the parent CIP, which was linked to their influence on the N-4 protonation capacity. The rise in LogPexp of compound X owing to the addition of the naphthyl moiety in comparison to CIP has a potential impact on enhancing the lipophilicity of these compounds, which may affect cell penetration [37].

One of the biological cellular mechanisms to DNA injury that preserves genomic in-tegrity is cell cycle arrest. Checkpoints aid in the monitoring of the sequence of events in the cell cycle and guarantee that subsequent cell cycle events occur only after the completion of the preceding one. Following DNA damage, cell cycle arrest occurs either in G1, S phase or before mitosis in the G2/M checkpoint [42]. Our data elucidated that CMB signifi-cantly increased cell proportion in pre-G1 in both OVCAR-3 and A-549 cell lines com-pared to untreated control cells. Cells’ accumulation in the pre-G1 phase might be the result of DNA fragmentation, suggesting that apoptosis may play a role in CMB-induced cancer apoptosis and cytotoxicity. Additionally, CMB induced a significant rise in the proportion of cells at S phases in OVCAR-3 and A-549 cells when compared to untreated control. This finding may result from S phase arrest, indicating a mechanism of topoisomerase II inhibition. According to our findings, the cell cycle blockage in the S phase caused by CMB contributed to the suppression of cancer cell proliferative activity by upregulating the expression of p53, which is consistent with the observations of Ude et al. [43]. Our observations are in contrast with Beberok et al., who elucidated that CIP can induce G2/M phase cell cycle arrest in melanoma cells [36]. As a result, depending on the cell origin and type, numerous pathways of drug action may predominate. Moreover, different fluoroquinolone derivatives may activate several diverse biochemical pathways within a single cell line [44].

Cancer cells can develop resistance to apoptosis via mutation or modulating the expression of proapoptotic proteins, including Bax, or by inducing anti-apoptotic protein expression, such as Bcl-2. Both Bax and Bcl-2 expression is regulated by the p53 tumor suppressor gene [45]. It is well established that the Bcl-2/Bax ratio is an essential predictor of apoptosis [46]. A drop in the Bcl-2/Bax index might result in MMP loss and improved mitochondrial membrane penetrability, allowing mitochondrial cytochrome c to leak inside the cytoplasm [47]. Following that, activated cytoplasmic cytochrome c activate caspase-9 and caspase-3, inducing PARP dissociation [48]. Therefore, the expression of numerous relevant apoptosis markers was evaluated first. Our data revealed that CMB inhibited the expression of Bcl-2 but increased p53, p21, Bax, and cleaved caspase-3 expression. It is evident that treatment with CIP inhibits tumor progression by triggering cell cycle arrest at the S phase and promoting cell death via p53 overexpression. This could subsequently lead to Bax overexpression and Bcl2 down expression, ultimately resulting in apoptosis. Therefore, it may stimulate the Bax/Bcl-2-dependent pathway to open the mitochondrial permeability transition pores [41]. These findings suggested that a mitochondrial apoptotic pathway could be implicated in HGSOC and NSCLC cells. Our results are consistent with Beberok et al., who showed that CIP dramatically raised p53 and Bax expression but suppressed Bcl-2 expression [41].

In a bid to gain more information regarding the mechanisms attenuating cell prolif-eration, the expression of the MAPK/ERK pathway has been evaluated. The implication of this signaling pathway in the development of lung cancer, particularly NSCLC, has been extensively recognized [24]. Growing evidence shows that oncogenic mutations (e.g., BRAF and RAS mutations) cause numerous alterations in the expression of genes implicated in cell cycle control, migration, differentiation, and cancer stem cells, all of these are important for NSCLC pathogenesis and treatment resistance [23,24]. Moreover, the MAPK/ERK signaling pathway is involved in the malignancy aspect of ovarian cancer [49,50], and ovarian cancer patients with altered MAPK pathways have been frequently documented [51]. It has been documented that the MAPK pathway gene copy number amplifications or mutations were associated with ovarian cancer chemical resistance [26,52]. Since the sustained triggering of MAPK/ERK signaling promotes the transition of normal cells into tumor cells, whereas attenuation of MAPK/ERK signaling restores non-transformed cancer cells in vitro and inhibits tumor development in vivo [53]. Our study hypothesized that CMB inhibited cell growth and tumor development of HGSOC and NSCLC in a time-dependent manner via modulating the MAPK/ERK signaling pathways, as demonstrated by inhibiting its downstream target ERK1/2. Thus, CMB could attenuate the MAPK/ERK signaling in NSCLC and HGSOC, which is consistent with Pandian et al., and Yu et al., who showed that fluoroquinolones could attenuate MAPK/ERK expression in cancer cell lines [54,55].

Since cancer may have hundreds of gene mutations and several molecular pathways that interact during tumor formation, it is highly unlikely that cancer can be controlled by targeting a single signaling pathway. As a result, chemoresistance may develop during therapy, which is a main cause of chemotherapeutics’ failure [56]. Therefore, the expression of the MAPK/JNK pathway has been evaluated. Aberrant JNK activation has been reported frequently in NSCLC [26,57], and a number of studies support the hypothesis that JNK has a favorable role in enhancing the growth of NSCLC as it promotes neoplastic transformation via negatively regulating p53 through c-Jun [58]. Additionally, it was elucidated that blocking the MAPK/JNK signaling pathway inhibited the proliferation of NSCLC [59]. These findings support our hypothesis that CMB slowed the growth of NSCLC via modulating the MAPK/JNK signaling pathway. 

Not only is JNK implicated in NSCLC, but it is also involved in ovarian cancer malignancy. The JNK pathway may be activated in the vast majority of ovarian cancers, most notably HGSOC [20,21]. Regardless of clinical stage, active JNK expression was adversely related to ovarian cancer patient survival and was higher in patients with late stages (III and IV) than in patients with early stages (I and II) [21]. This suggests that JNK pathway activity may rise with disease progression and deleteriously impact ovarian cancer patients’ clinical outcomes. This led us to the conclusion that ovarian tumors become chemo-resistant not just because they are treated with chemotherapeutics but also because they propagate to a more aggressive stage with elevated JNK activity over time throughout their normal clinical course. This hypothesis supports our findings that CMB could be a potential candidate for managing HGSOC progression via attenuating the MAPK/JNK signaling pathway. Our results are in agreement with Werber et al., who elucidated that fluoroquinolone could inhibit JNK expression in human cancer cell lines [60]. Taken together, our data clearly illustrated that CMB suppressed HGSOC and NSCLC proliferation and cell cycle progression by targeting the MAPK pathway, p53/p21, and p53/Bax/Bcl-2 signaling pathways.

## 4. Materials and Methods

### 4.1. Chemistry

**Figure 8:**
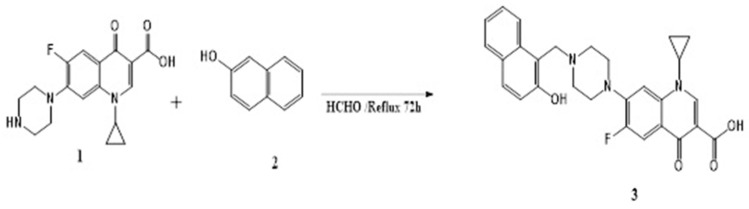
Synthesis of novel CMB [37].

Phenolic C-Mannich base 3 was prepared by refluxing CIP base 1 and 2-naphtol 2 with excess formaldehyde 37% in ethanol (Figure 8) [37]. The formed precipitate filtered and recrystallized from aqueous ethanol. Regarding the spectral data, 1H NMR chart showed the newly formed methylene group of Mannich base (C-CH2-N) as a singlet of 2 protons at chemical shift δ = 4.4 ppm, along with the common pattern of the ciprofloxacin moiety including the eight piperazine protons as two multiplets in the range 2.7–3.4 ppm; the signal at range of δ 6.8–7.25 ppm appeared as doublet with JH–F of about δ 6.6–7.5 Hz ppm assigned as H-8; the other doublet that appeared at the range of δ 7.8–8.1 ppm with JH–F of about δ 12.0–16 Hz assigned as H-5 and the singlet signal characteristic for H-2 at about δ 8.63–8.95 ppm. The 13C-NMR showed the characteristic signal at δ of δ 56 ppm, which corresponds to C-CH2-N- methylene of Mannich base. The purity of the targeted compounds was checked and confirmed by elemental analysis [37] (See Supplementary Materials).

1-Cyclopropyl-6-fluoro-7-(4-((2-hydroxynaphthalen-1-yl) methyl) piperazin-1-yl)-4-oxo-1,4-dihydroquinoline-3-carboxylic acid CMB.

Yellowish white powder; m.p: 236 °C–237 °C; Yield = 72%; 1H NMR (400 MHz, DMSO-d6) δ (ppm): 15.18 (1H, s, COOH), 10.67 (1H, s, OH), 8.63 (1H, s, H-2), 7.86 (1H, d, J H-F = 13.2 Hz, H-5) 7.55 (1H, d, J = 8.0 Hz, H-8), 8.05 (1H, d, ArH), 7.78 (1H, d, J = 8.0 Hz, ArH), 7.72 (1H, d, J = 8.0 Hz, ArH), 7.47–7.45 (1H, m, ArH),7.29 (1H, t, J = 8.0 Hz, ArH), 7.14 (1H, d, J = 8.0 Hz, ArH), 4.10 (2H, s, N-CH2 Mannich base), 3.78–3.77 (1H, m,-cyclopropyl CH), 3.46–3.44 (4H, m, piperazine 4H), 2.78–2.76 (4H, m, piperazine 4H),1.31–1.30 (2H, m, cyclopropyl CH2), 1.16–1.14 (2H, m, cyclopropyl CH2);13CNMR (100 MHz, DMSO-d6) δ (ppm): 176.82, 166.44, 155.04, 152.29 (JC-F = 275 Hz),148.47, 145.60, 139.62, 134.01, 129.36, 128.60, 126.62, 122.89, 119.19, 113.54, 111.48 (JC-F = 23 Hz), 107.18, 53.23, 52.45, 49.95, 36.35, 8.03. Anal. Calcd. For C28H26FN3O4 (487.52) C, 68.98; H, 5.38; F, 3.90; N, 8.62; O, 13.13 Found C, 69.17; H, 5.45; N, 8.81.

### 4.2. Cell Lines and Cell Culture

OVCAR-3 and A-549 cells were provided from American type culture collection (ATCC, Manassas, VA, USA). They were cultured at 37° in fresh Dulbecco’s Modified Eagle’s Medium (DMEM, Sigma-Aldrich, Inc, St Louis, MO, USA) enriched with 10% fetal bovine serum (FBS, Biosolutions International, Melbourne, Australia), 1% penicillin-streptomycin mixture (Invitrogen, Grand Island, NY, USA), and 1% L-glutamine (Sigma-Aldrich, Inc) in a humidified environment containing 5% carbon dioxide.

### 4.3. Cell Viability Assay

MTT assay was performed to assess the viable OVCAR-3 and A-549 cells following treatment with CMB and doxorubicin. All growing cells were cultured in triplicate in ninety-six well plates at a density of 1 × 10^4^ cells/well and allowed to proliferate for twenty-four hours in fresh DMEM media. Cells were treated with serial dilutions of CMB or doxorubicin incubated for 48 h at 37  °C in a 5% CO_2_ incubator. Next, 20 microliters of MTT (5 mg/mL in PBS) reagent were added to each well and incubated for four hours in dark. For formazan crystals’ solubilization, 100 μL of DMSO was used. ELISA plate reader (Model 550, Bio-Rad, Hercules, CA, USA) was used to estimate the optical density at 570  nm. Cell viability at numerous concentrations was estimated relative to that of untreated cells [61]. For both OVCAR-3 and A-549 cell lines, the optimum IC_50_ values were obtained from the dose-response curve.

### 4.4. Annexin V Apoptosis Assay

Apoptosis was evaluated using the Annexin V-FITC apoptosis detection kit, as directed by the manufacturer (Immunotech, Marseille, France). The annexin V-FITC kit, in conjunction with flow cytometry, allows for the quantification of apoptotic cells. This procedure, using Annexin V/PI double staining, allows us to assess the translocation of phosphatidylserine to the cell membrane’s outer leaflet. Additionally, annexin V binds to phosphatidylserine in late apoptotic cells, but as their membrane integrity has been disrupted, they may be recognized from early apoptotic cells using PI. 1 × 10^5^ cells were incubated for 48 h with tested drugs before being collected into falcon tubes. The cells were rinsed with PBS twice after centrifugation. The cell residue was resuspended in binding buffer, then Annexin V (5 µL) and PI (10 mL) were added in 500 µL of cell suspension and incubated for thirty minutes at 4 °C in dark. To estimate the fraction of apoptotic cells, 10,000 cells from each sample were detected using FACS Calibur flow cytometer [62].

### 4.5. Cell Cycle Analysis

1 × 10^6^ cells were cultured into six-well plates for twenty-four hours; old medium was removed, and the medium containing tested drugs (IC_50_) was added. The cells were collected and washed with phosphate buffer saline (PBS, pH = 7.4, Sigma-Aldrich, Inc, St. Louis, MO, USA) after 48 h of incubation. 70% cold ethanol was added for cell fixation before keeping at 4 °C overnight. Then, the cells were rinsed with PBS and incubated for thirty minutes with 1 mg/mL RNase before being stained with PI (50 μg/mL), which was used to label the intracellular DNA by incubating the cells for at least 20 min at 4 °C, and finally, flow cytometry was used to analyze the cells (BD FACS Calibur) [63,64].

### 4.6. RNA Isolation and Real-Time qPCR Assay

5 × 10^5^ cells were cultivated in six-well plate in triplicate. Cells were cultivated in DMEM medium and incubated at 37 °C in a humidified carbon dioxide atmosphere. Cells were treated with CMB and DOX (IC_50_) for 24 or 48 h. Trizol reagent (Invitrogen, Waltham, MA, USA) was utilized to extract total RNA according to the manufacturer’s instructions. Briefly, cells were incubated in one ml of Trizol reagent, 200 microliter chloroform was added and centrifuged at 1400× *g* for fifty minutes at 4 °C. The upper aqueous layer was transferred to a new tube, and RNA was precipitated by using 0.5 mL of ice-cold isopropanol. RNA pellet was rinsed with ethanol and resuspended in DEPC water [65]. RNA yield and purity were estimated using Nanodrop 1000 (Thermo Scientific, Waltham, MA, USA) [66]. All isolated RNA was reverse-transcribed via the High-capacity cDNA Reverse Transcription Kit according to the manufacturer’s instructions. The resulting cDNA was subjected to qRT-PCR using maxima SYBR green master mix (thermos-scientific, Waltham, MA, USA). Step one Real-Time PCR system was utilized to evaluate the gene expression of p53, p21, Bax, and Bcl-2. Each sample was carried out in triplicate [67]. The PCR thermal cycling settings were as follows: 95 °C for ten minutes for amplification activation and 40 cycles at 95 °C for fifteen seconds, 60 °C for thirty seconds, and 72 °C for thirty seconds. The GAPDH gene was employed as an internal control. NBCI was used to design forward and reverse primer sequences for the genes GAPDH, p53, p21, Bax, and Bcl-2 (Table 2). The results were analyzed using the relative quantitation method, which used the Delta-Delta-Ct (∆∆Ct) Algorithm to estimate relative gene expression in comparison to untreated cells [68].

### 4.7. Western Blotting Assay

Before preparing cell lysates, cells (2 × 10^5^) were treated for 24 or 48 h with CMB or DOX (IC_50_). Cell lysis buffer, RIPA (120 mmol/L NaCl, 20 mmol/L Tris–HCl, pH 7.5, 0.1% SDS, 1.0% Triton X-100, 10% glycerol, 1 mmol/L EDTA and 1% sodium deoxycholate), and proteinase inhibitor were added. The ReadyPrepTM protein extraction kit (Bio-Rad Inc., cat#163-2086, Hercules, CA, USA) was added to each sample of cell pellet to extract proteins as directed by the manufacturer. The Bradford assay (SK3041) was performed to determine protein concentration in each sample and was provided by Bio basic Inc (Markham Ontario L3R 8T4 Canada). The supernatant was denatured at 100 °C for 10 min after being mixed with protein loading buffer. SDS-PAGE was used to separate equivalent quantities of protein extracts (20 µg), which were then electrophoretically transferred onto PVDF membranes. The membrane was blocked before incubation with the specified primary antibodies (dilution 1:500): t-ERK1/2, p-ERK1/2, t-JNK1/2, p-JNK1/2, t-MKK4, p-MKK4, t-MKK7, p-MKK7, cleaved caspase-3 and GAPDH at 4 °C overnight. Blots were properly washed before being incubated with HRP-conjugated secondary antibody Goat anti-rabbit IgG (Novus Biologicals) (dilution 1:5000) for 1 h. Finally, blots were evaluated using an enhanced chemiluminescence substrate (Clarity TM Western ECL substrate Bio-Rad cat#170-5060). A CCD camera-based imager was used to record the chemiluminescent signals. (LAS-400, Fujifilm Co., Tokyo, Japan). Image J software was finally used to read the band intensity of target proteins. Band density was normalized relative to beta-actin (housekeeping protein) [69].

### 4.8. Statistical Analysis

The data were analyzed using GraphPad Prism 7.0. (GraphPad Software, La Jolla, CA, USA). The data were presented as a mean ± SEM. To analyze the differences between studied groups, the one-way ANOVA test was used, followed by Tukey’s post hoc test. The significance level was set at *p* < 0.05.

## 5. Conclusions

CMB displayed marked cytotoxicity against ovarian and lung cancer cells. Mecha-nistically, CMB showed anti-proliferative action towards OVCAR-3 and A-549 cancer cells through inducing apoptosis via a mitochondrial apoptotic pathway mediated by P53/Bax/Bcl-2 and caspase-3 along with arresting cell cycle at S phase via modulating the MAPK signaling pathway. Accordingly, CMB could be considered a promising anti-cancer agent for both lung and ovarian cancers. Further in vivo work is needed to confirm the current data.

## Figures and Tables

**Table 1:** Distribution of apoptotic cells in the OVCAR−3 and A−549 cell lines following CMB and Doxorubicin treatment, as assessed by the Annexin V-FITC assay.

Code	Apoptosis			Necrosis
	Total	Early	Late	
CMB/OVCAR-3	36.26	3.22	23.42	9.62
Dox/OVCAR-3	43.62	2.99	26.16	14.47
Cont/OVCAR-3	2.92	0.53	0.21	2.18
CMB/A-549	33.61	4.88	17.65	11.08
Dox/A-549	42.51	1.61	25.78	15.12
Cont/A-549	1.71	0.39	0.14	1.18

**Table 2:** Sequences of primers.

Primer		Sequence
*P53*	Forward	5′- GGTGACACGCTTCCCTGGAT-3′
	Reverse	5′- CATCCATTGCTTGGGACGGC-3′
*P21*	Forward	5′- GAGCAGCTGCCGAAGTCAGT-3′
	Reverse	5′- CGCCATTAGCGCATCACAGT-3′
*Bax*	Forward	5′- CTGCAGAGGATGATTGCCGC-3′
	Reverse	5′-GGGCGTCCCAAAGTAGGAGA-3′
*Bcl2*	Forward	5′- CTGGTGGACAACATCGCCCT-3′
	Reverse	5′-GCCGTACAGTTCCACAAAGGC-3′
*GAPDH*	Forward	5′-CGGGGCTCTCCAGAACATCAT-3′
	Reverse	5′-GTCCACCACTGACACGTTGG-3′

## Data Availability

Data generated or analyzed during this study are provided in full within the published article and its Supplementary Materials.

## Supplementary Materials

The following supporting information can be downloaded at: https://www.mdpi.com/article/10.3390/molecules28031137/s1, Figure S1: H1NMR spectrum of CIP mannich base (400 MHz, DMSO-*d6*). Figure S2: 13C-NMR spectrum of CIP mannich base (400 MHz, DMSO-*d6*).

## Sample Availability

Samples of the compounds are available from the authors.
