# Supplementary material for: In Vitro Anticancer Activity of Novel Ciprofloxacin Mannich Base in Lung Adenocarcinoma and High-Grade Serous Ovarian Cancer Cell Lines via Attenuating MAPK Signaling Pathway
Source: Molecules. 2023 Jan 23;28(3):1137. doi: 10.3390/molecules28031137 (PMC9921546; doi:10.3390/molecules28031137)
Supplement: Supplementary file 1 [file molecules-28-01137-s001.zip › molecules-2107192-supplementary.pdf]

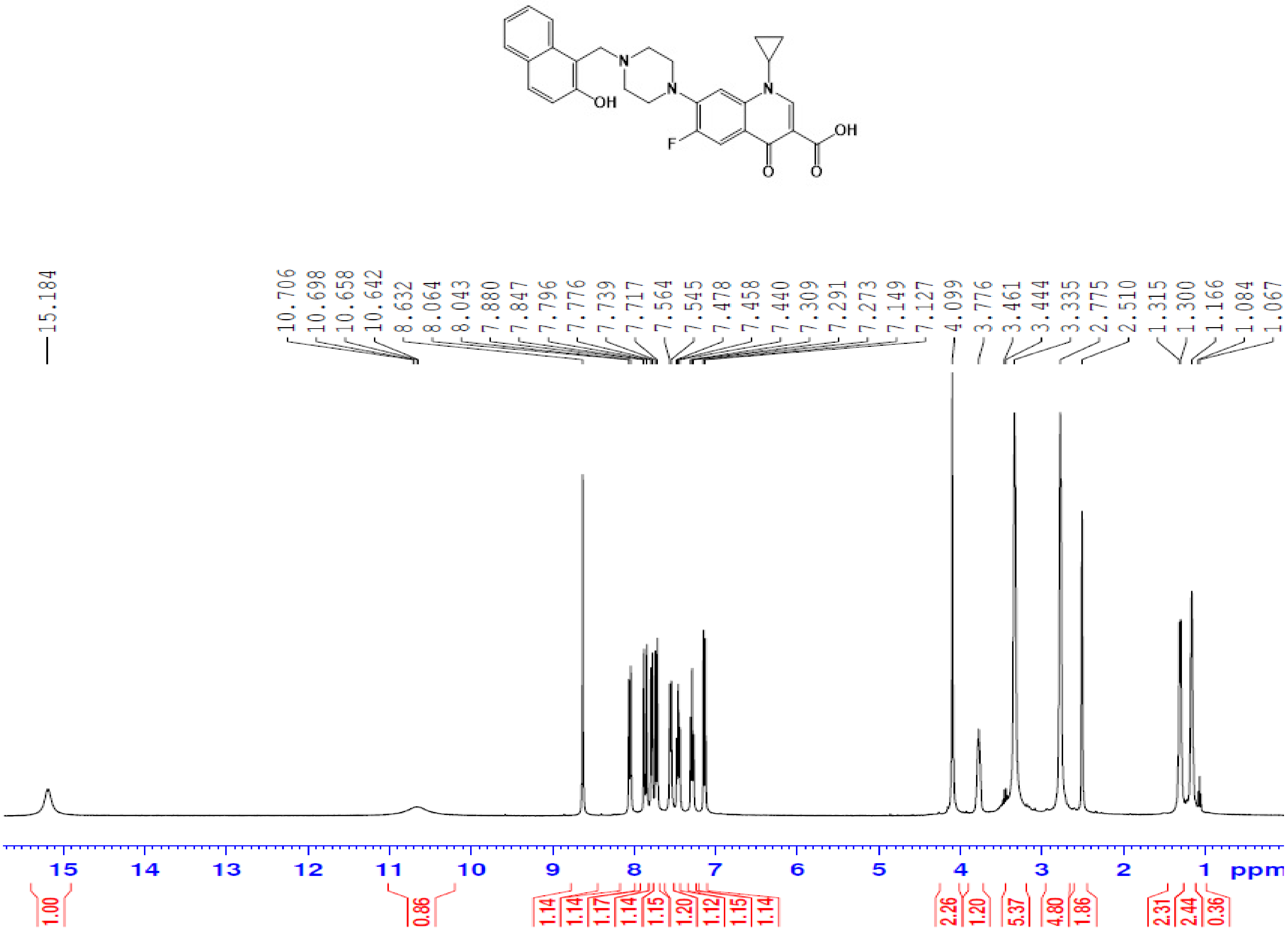

Figure S1: H1NMR spectrum of CIP mannich base **(400 MHz, DMSO-*d6*).**

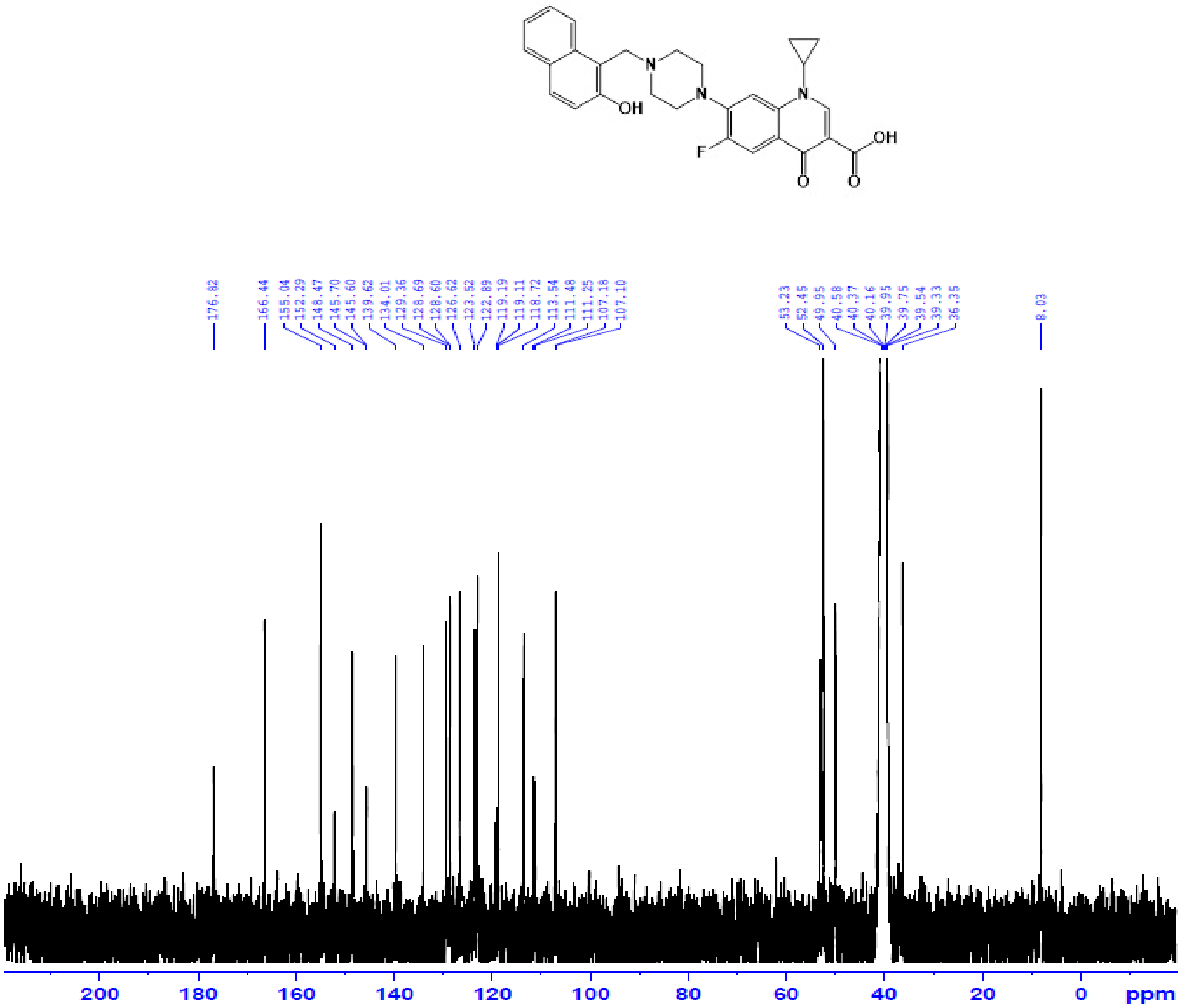

Figure S2: ^{13}C-NMR spectrum of CIP mannich base **(400 MHz, DMSO-*d*6).**
